# Post-Operative Safety Outcomes and 5-Year Healthcare Utilisation After Bariatric Surgery vs. Other Elective Types of Surgical Treatments – A Retrospective Observational Cohort Study

**DOI:** 10.1007/s11695-026-08584-7

**Published:** 2026-04-11

**Authors:** Ghassan Chamseddine, Robert David McIntyre, Spyros Panagiotopoulos, A Caroline Rudisill, James Casella, Lidia Castagneto-Gissey, Avril Chang, Gabriele Galata, Amyn Haji, Asif Haq, Laurent Genser, Geltrude Mingrone, Ameet Patel, Angelo Salerno, Daniele Tassinari, Francesco Rubino

**Affiliations:** 1 Department Bariatric/Metabolic & Minimally Invasive Surgery, King’s College Hospital NHS Foundation Trust, London, UK; 2 School of Sport, Exercise & Applied Science, St Mary’s University Twickenham London, London, UK; 3 Department of Health Promotion Education & Behavior, Arnold School of Public Health, University of South Carolina, Columbia, USA; 4 Department of General Surgery, Ospedale dei Castelli, Rome, Italy; 5 Department of Surgical Sciences, Sapienza University of Rome, Rome, Italy; 6 Department of Endocrine & General Surgery, King’s College Hospital NHS Foundation Trust, London, UK; 7 Department of Colorectal Surgery, King’s College Hospital NHS Foundation Trust, London, UK; 8 Hepato-Biliary & Pancreatic Surgery, Pitié-Salpêtrière University Hospital, Sorbonne University Assistance Publique-Hôpitaux de Paris, Paris, France; 9 Catholic University of the Sacred Heart, Milan, Italy; 10 School of Cardiovascular & Metabolic Medicine & Sciences, King’s College London, London, UK; 11 IRCCS Ospedale Galeazzi Sant’Ambrogio, Milan, Italy

## Abstract

**Objective:**

To assess safety and healthcare utilization (HU) after metabolic/bariatric surgery (MBS) and compare it to commonly performed general surgery procedures for benign indications.

**Research Design and Methods:**

Data obtained from a nationwide database (NHS Digital) on 800 consecutive patients who underwent bariatric surgery (*n* = 100) and seven other types of surgery (*n* = 100 for each) for non-acute, non-cancer indications at a tertiary hospital in England. Non-MBS procedures included adrenalectomy, gastro-esophageal junction surgery (GEJ), in-patient cholecystectomy (IC), outpatient cholecystectomy (OC), colorectal surgery (CR), hernia surgery (HS) and neck endocrine surgery (NES).

**Results:**

Despite a higher number of preoperative co-morbidities and ASA score, MBS had significantly lower odds of 30-day major complications (Clavien–Dindo grade≥IIIa) compared to CR (6%;*p* = 0.013) and GEJ (8%;*p* = 0.004). No differences in complication rates with all other groups. The total number of procedure-related readmissions over 5-year after MBS were similar to HS, NES or IC and lower than CR, AS, and GEJ. The average per-patient 5-year cost of procedure-related readmissions after MBS was £790 + 337, lower than for CR (£8,270 + 3218, *p* < 0.001), AS (£3242 + 772, *p* < 0.001) and GEJ (£1705 + 976, *p* = 0.013) and similar to other procedures. There was a higher number of outpatient visits after MBS in the first two years after surgery, consistent with national protocols and no differences in 5-year A&E attendance among groups.

**Conclusions:**

MBS is associated with a relatively low risk of post-operative complications and 5-year healthcare use compared to other commonly used types of surgical treatments. These findings could help dispel misperceptions that undermine access to effective treatment of obesity.

## Introduction

Bariatric surgery is the most effective treatment for severe obesity [1]. It is also a standard treatment for type 2 diabetes, a practice referred to as metabolic surgery [2, 3].

Despite its strong safety profile, with mortality rates of 0.1–0.8% and major complication rates of 2–6%, comparable to common elective surgeries—it remains underutilized, with only 0.3–2.0% of eligible patients accessing it [4–7]. Although metabolic/bariatric surgery (MBS) is cost-effective [8, 9] concerns over risks and long-term costs persist [10, 11].

Misconceptions—often amplified by media—contribute to hesitation among physicians and patients, and may limit insurance coverage [12–15]. These concerns contrast with broader acceptance of other elective surgeries, which are more frequently performed despite treating less prevalent conditions. [16–18] Studies show that concerns about safety undermine both physicians’ referral to and patients’ acceptance of MBS [19]. Also, despite evidence of cost-effectiveness [8, 20, 21], there are concerns that MBS may increase long-term healthcare use for several years post-operatively [20, 22], contributing to inadequate or total lack of insurance coverage for bariatric surgery in some countries [23, 24]. 

There seem to be less apprehension about the safety and costs of other types of surgery. (i.e., gallbladder surgery, colectomy, knee, and hip replacement) which are performed more often than MBS, despite addressing less prevalent diseases.

Although previous reports have suggested that the risk of major complications after MBS may be similar to other commonly performed operations [6, 7], such conclusions are based on data derived from unrelated case-series, and short-term safety outcomes. No study has contextually investigated the long-term healthcare utilization (HU) associated with complications of MBS relative to other elective surgeries. Such contextualisation could enable a more balanced assessment of MBS’s risks and benefits, supporting informed decisions by patients, physicians, and policymakers.

In this study we aimed to investigate the safety, 5-year HU and costs of MBS compared to other elective procedures in general surgery for non-acute benign conditions.

## Methods

### Aim and Design

This observational study was approved by the Research ethics committee and the Confidentiality Advisory Group (CAG), UK. Public opinion in the design of the study was obtained as part of CAG application requirement prior to approval. The aim was to assess the peri-operative (30-day) safety profile, post-surgery healthcare usage, and related costs across cohorts of patients undergoing common elective general surgery procedures.

The following procedures were assessed: Adrenal surgery (AS), Gastro-Esophageal Junction surgery (GEJ), MBS, In-patient Cholecystectomy (IC), Out-patient Cholecystectomy (OC), colorectal procedures (CR), Hernia (HS) and Neck-endocrine surgery (NES). IC and OC differ in safety outcomes, 100 patients of each were included and analysed separately. Emergency operations and operations for malignancy were excluded. (Appendix 1 lists operations and indications.)

Patients for MBS were identified from a prospectively gathered database. 100 consecutive adult elective patients (all newcomers) from 2014 to 2015 were selected and all operated by a specialised and experienced consultant bariatric surgeon with more than 10 years’ experience in high volume bariatric centre. Bariatric surgery included revisional and primary procedures (sleeve gastrectomy and Roux-en-Y gastric bypass accounted for > 90% of patients). Emergency MBS patients were excluded. Patients for other surgeries were the last 100 consecutive non-acute patients operated for benign conditions before 2015 by specialist consultant surgeons at the same tertiary-care hospital with similar level of experience. Emergency and cancer cases were excluded.

To reflect usual clinical practice and minimize bias, we included in this study an equal number of unselected, consecutive patients who underwent any type of standard procedure for each surgical subspecialty. Since baseline disease status and typical demographics of surgical candidates contribute to the safety profile and cost impact of surgical specialties, we intentionally did not match for baseline characteristics.

Peri-operative safety (inpatient and 30-day outcomes) and 5-year HU and costs were investigated for each sub-cohort of patients.

### Data Sources and Study Outcomes

For the index operation, patient demographics, baseline surgical risk (American Society of Anaesthesiologists score [ASA] score), hospital stay, operation details, inpatient and 30-day complications were obtained from hospital records.

Healthcare usage over the 5-year period (including 30-day postoperatively), was assessed via an independent national administrative database (NHS-Digital). Using patient’s unique NHS numbers, NHS digital provided data related to all hospital-related encounters that occurred anywhere in England, including inpatient admissions, emergency room attendance and outpatient visits for each patient. Each healthcare encounter (episode) included dates, diagnoses (ICD10 codes), and procedures (OPCS4 codes), if any.

Severity of postoperative complications was classified using the Clavien–Dindo system [25]. “Major complications” were defined as Clavien–Dindo grade ≥ IIIa.

To assess whether the diagnosis of each episode was related to the index procedure, clinical description of the primary diagnosis codes (ICD10) was presented to consultants with expertise in each subspecialty. They were asked to adjudicate whether diagnoses could represent complications and/or sequelae related to the index procedure.

Most hospital encounters included a procedure or intervention described using OPCS4 codes. As with the ICD10 diagnosis codes, the clinical description of these procedure/interventions was presented to the same consultants to determine if related to the index operation.

Costs were calculated using HRG reimbursement tariffs, reflecting hospital compensation. Assistance from the coding and costing department at the Hospital where the operation happened was sought to determine the reimbursement amount associated with each HRG code in the respective year. Costs for hospital-based care not undertaken at the initial hospital were assumed to have the same reimbursement level. Costs were calculated for accident and emergency (A&E) attendance, outpatient visits and inpatient admissions for all hospital-based care occurring nationwide. The database does not include visits in primary care practice or community services.

### Statistical Methods

Data was analysed using SPSS version 28 (IBM Corporation, NY, USA) or GraphPad Prism Version 9.4 (GraphPad Software Inc. La Jolla, USA). Data are reported as mean ± 95% confidence interval (CI), unless otherwise stated. Statistical significance was accepted at *P* < 0·05. Patient demographics, baseline surgical risk, length of stay (LoS), inpatient and 30-day postoperative complications, readmissions and cost were compared between specialties using the Kruskal-Wallis test with Mann-Whitney *post hoc* comparisons. The proportion of patients that were readmitted following surgery was compared between specialties using binary logistic regression with age, gender, ASA score, and number of comorbidities as covariates. The proportion of patients with major complications and patient reoperation rates following surgery were compared between procedures using the Chi-Square test. Bonferroni-adjusted pairwise comparisons were conducted where appropriate to detect differences between bariatric surgery and comparator surgeries. Differences in readmissions and cost between procedures were confirmed on log-plus-one-transformed data using a one-way analysis of covariance (ANCOVA), with age as a covariate, and Bonferroni-adjusted pairwise comparisons where appropriate.

## Results

### Study Population

The study included 800 patients. Demographics and peri-operative outcomes are reported in Table 1. All MBS procedures were performed between 2014 and 2015. More than 90% of non-bariatric procedures were performed between 2013 and 2015.

**Table 1:** Patient demographics and operative outcomes

	Bariatic	Adrenal	GEJ	Colorectal	Inpatient cholecystectomy	Lap hernia	Neck surgery	Outpatient cholecystectomy
Female gender (%)	75	59	55*	49**	68	48**	87	87
Age (years)	47±3	53±3**	54±3**	49±3	54±3**	59±3**	53±3*	45±3
Number of comorbidities	3±0	2±0**	2±0**	2±0**	2±0**	2±0**	2±0**	1±0**
ASA score	3±0	2±0**	2±0**	2±0**	2±0**	2±0**	2±0**	1±0**
Operative time (min)	129±8	129±11	135±10	227±28**	90±7**	91±8**	90±5**	92±8**
Length of stay (days)	2.3±0.3	3.9±0.6**	4.2±1.4**	12.2±1.9**	2.4±1.9*	1.9±0.3*	2.7±1.4**	0.4±0.3**
Inpatient major complications (%)	0	1	7**	8**	4	1	1	1

Data are represented as means ± 95% confidence intervals, or percentages

*ASA* American society of anaesthesiologists

**denotes different from bariatric surgery, P < 0.01; *denotes different from bariatric surgery, P < 0.05

MBS patients were younger (p≤0·021) but had a significantly higher number ofco-morbidities (p≤0·042) and higher ASA score (p<0·001) than other groups,indicating substantially higher surgical risk and disease-burden at baseline.

### In-patient Peri-operative Outcomes

Operative time for MBS was shorter than for CR (*p* < 0·001) but longer than IC and OC, HS, and NES (*p* < 0·001). Hospital stay for MBS (2·3 *±* 0·3 days) was shorter than for IC (2·4 *±* 1·9; *p* < 0·05), NES (2·7 *±* 1·4; *p* < 0.001), AS (3·9 *±* 0·6; *p* < 0·001) and CR (12·2 *±* 1·9; *p* < 0·001) but longer than for OC (0·4 *±* 0·3; *p* = 0·034) and HS (1·9 *±* 0·3; *p* = 0·018). The odds of having a major inpatient complication (Clavien–Dindo grade ≥ IIIa) during the index hospital admission was lower in MBS (no major complications), compared to CR (8%; *p* = 0·026) and GEJ surgery (7%; *p* = 0·038), whereas no statistically significant differences were observed compared with other groups.

### Short-term (30-day Post-surgery) Post-operative Safety Outcomes and Costs

30-day safety outcomes and cost are shown in Table 2. MBS surgery was associated with a significantly lower rate of 30-day major complications (no major complications) compared to GEJ (8%; *p* = 0·004) and CR (6%; *p* = 0·013); no differences were detected with other groups. There were no reoperations within 30-day postoperatively in the MBS and AS whereas the reoperation rate ranged between 1% and 4% among other groups. This was not statistically significant. MBS’s patients were less likely to require hospital readmission for any cause within 30-day postoperatively (7% overall readmission rate) compared with CR (21%; *p* = 0·039) and AS (26%; *p* = 0·013), whereas no statistically significant difference was observed compared with other groups. MBS was also associated with a significantly lower rate of 30-day procedure-related readmissions (4%), compared to AS (14%; *p* = 0·041) and CR (15%; *p* = 0·031). No differences were observed with other groups.

**Table 2:** 30-day outcomes and cost

		Bariatric	Adrenal	GEJ	Colorectal	Inpatient cholecystectomy	Lap hernia	Neck	Outpatient cholecystectomy
Morbidity	Major morbidity (%)	0	1	8**	6*	2	1	1	1
Reoperation rate (%)	0	0	0	4	1	1	1	1	1
Readmission rate	All causes (%)	7	26 *	13	21 *	10	7	8	8
	Procedure-related (%)	4	14 *	7	15 *	7	3	2	5
Readmissions Length of stay	All causes (days)	0.3 ± 0.4	1.1 ± 1.0	1.9 ± 1.0 **	2.6 ± 1.9**	1.9 ± 1.1 **	1.8 ± 2.5	5.1 ± 2.3 **	1.1 ± 1.1
	Procedure-related (days)	1.0 ± 1.2	1.0 ± 0.9	2.4 ± 1.5	3.6 ± 2.6	2.4 ± 1.2	1.0 ± 0.8	3.7 ± 3.8	1.7 ± 1.8
Total cost for all re-admissions	All causes (£)	10,067	64,651	25,624	51,186	16,599	16,212	40,618	12,954
	Procedure-related (£)	9,405	39,345	15,980	41,027	14,142	10,532	2,180	6,681
Cost per re-admission	All causes (£)	719 ± 734	1,658 ± 351**	1,602 ± 657**	1,765 ± 523**	1,037 ± 256**	1,474 ± 664*	2,389 ± 664**	1,295 ± 504*
	Procedure-related (£)	2,351 ± 2,236	1,874 ± 570	1,776 ± 968	2,051 ± 703	1,088 ± 312	1,505 ± 593	1,090 ± 6,772	1,114 ± 706

Mean Data are represented as means ± 95% confidence intervals

**denotes different from bariatric surgery *P* < 0.01, *denotes different from bariatric surgery *P* < 0.05

The mean length of stay for all-cause readmissions within the 30-day postoperative period was significantly lower for MBS (0·3 *±* 0·4 days) compared to GEJ (1·9 ± 1·0; *p* = 0·003) and CR (2·6 ± 1·9; *p* = 0·005), IC (1·9 ± 1·1 ; *p* = 0·015) and NES (5·1 ± 2.3 ; *p* < 0·001). A similar trend was observed in relation to the length of stay for procedure-related readmissions, however these differences were not statistically significant.

MBS was associated with the lowest total costs for all-cause 30-day readmissions, although differences with other procedures did not reach statistical significance.

There were no statistically significant differences in both total costs and cost-per-admission related to 30-day procedure-related readmissions among the various types of procedures (all *p*≥ 0·130).

We also measured attendance of accident and emergency services (A&E) within 30-day after the index surgery and related costs (Table 3, Appendix 2). Available diagnostic information (e.g., ICD10 codes) related to A&E room attendance did not allow for accurate attribution of the episode to the index procedure. However, it can be assumed that most visits to A&E room within the first 30-days after major surgery are likely to be associated with the index surgical procedure. There were no statistically significant differences in the total number or total cost of 30-day A&E visits. However, the cost per accident and emergency room visits after MBS (£79 *±* 17) was lower compared to CR (£112 *±* 14, *p* = 0·028) and OC (£121 *±* 21, *p* = 0·014), and similar compared to all other types of surgery (*p*≥0.079).

### Long-term (5-year Post-surgery) Outcomes

#### Hospital Readmissions

**Fig. 1:**
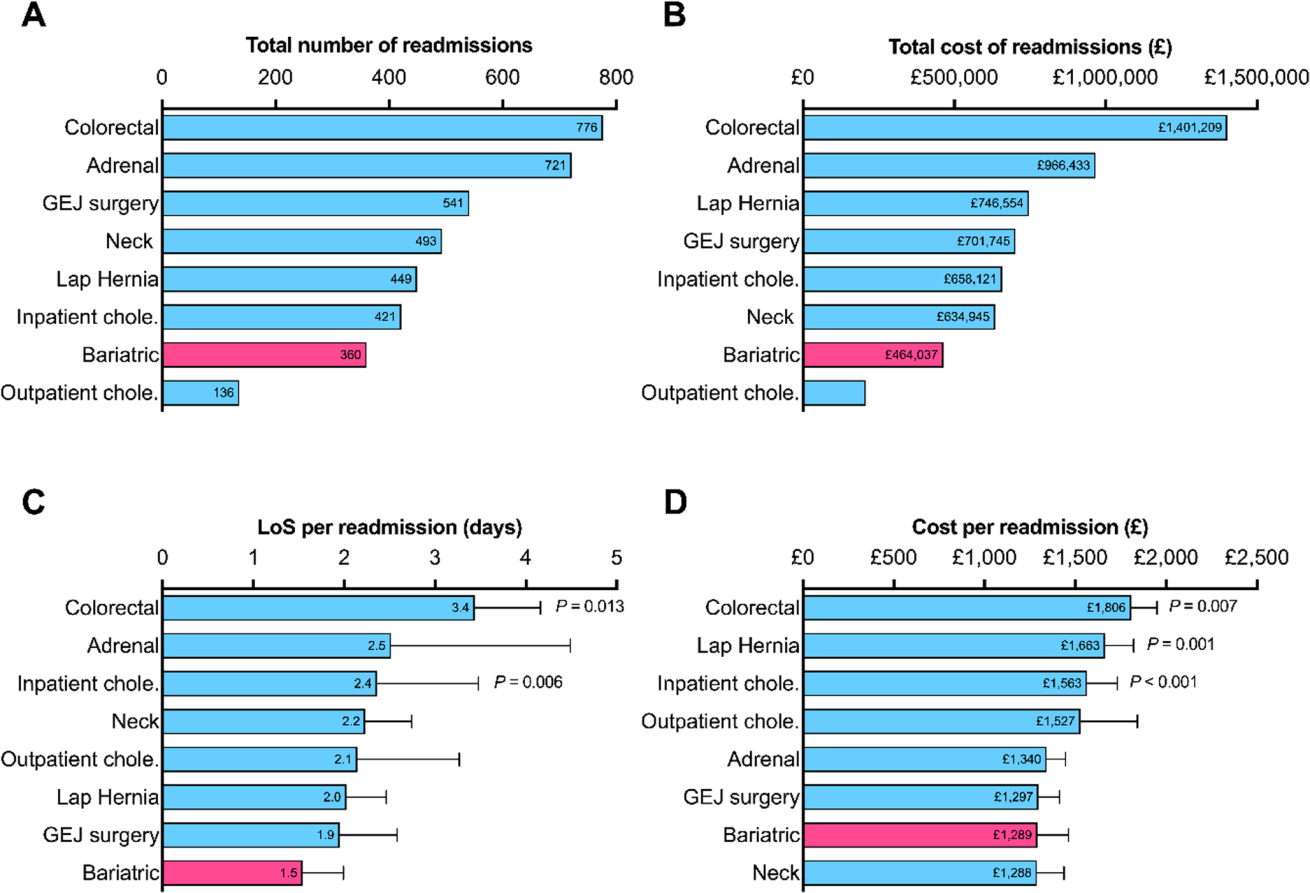
Total number (**A**), total cost (**B**), mean length of hospital stay (LoS) (**C**), and mean cost (**D**) of readmissions over the 5 years post-surgery in each operation type (*n* = 100 in each). Tails represent 95% confidence intervals. Analysed by the Kruskal-Wallis test with Bonferroni-adjusted pairwise comparisons (Mann-Whitney). P-values show statistically significant differences between bariatric surgery and comparator surgery

A total of 3,897 inpatient readmissions occurred over the 5-year period in all groups. The total number of inpatient readmissions (Fig. 1A) and associated cost (Fig. 1B) tended to be lower after MBS compared to all other types of surgery except for OC, although differences among groups did not reach statistical significance. Readmissions after MBS required shorter hospital stay (1·5 ± 0.5) compared to CR (3·4 ± 0.7, p = 0·013) and IC (2·4 ± 1·1, p < 0·001); Fig. 1C). In addition, the cost per readmission after MBS (£1289 ± 173) was lower than for CR (£1806 ± 142, p = 0·007), HS (£1663 ± 157, p = 0·001), and IC (£1563 ± 167, p < 0.001; Fig.1D).

**Fig. 2:**
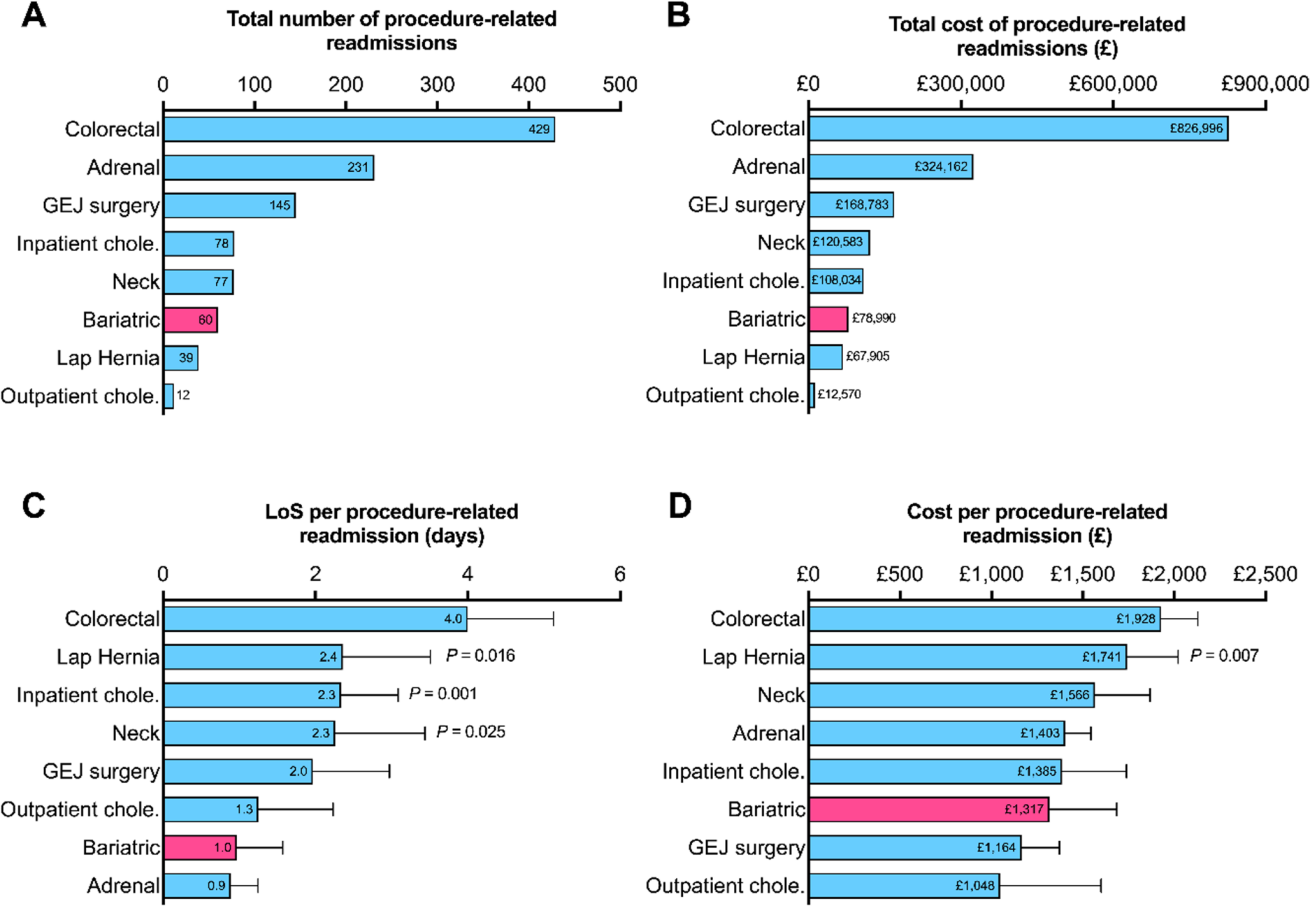
Total number (**A**), total cost (**B**), mean length of hospital stay (LoS) (**C**), and mean cost (**D**) of procedure-related readmissions over the 5 years post-surgery in each operation type (*n* = 100 in each). Tails represent 95% confidence intervals. Analysed by the Kruskal-Wallis test with Bonferroni-adjusted pairwise comparisons (Mann-Whitney). P-values show statistically significant differences between bariatric surgery and comparator surgery

Of the total 3,897 inpatient readmissions over 5-years, 1,071 (27%) were deemed procedure-related. The risk of patients being readmitted for a procedure-related issue was different between specialities (p< 0.001). Post hoc pairwise chi-square analyses revealed that procedure-related readmission was lower after MBS (31%) compared to CR (78%, p<0·001), AS (69%, p<0·001),and GEJ (54%, p=0·007). Conversely, MBS patients were at higher risk of a procedure-related readmission compared to OC (10%, p=0·002). There were no differences observed between MBS and HS (23%, p=1·00), NES (22%, p=1·00),or IC (21%, p= 0·749). It follows that the total number of procedure-related readmissions (Fig. 2A) and costs (Fig. 2B) after MBS was considerably lower than after CR, AS, and GEJ surgery.

The average LOS per procedure-related readmission (Fig. 2C) was shorter after MBS (1+0·6 days) compared to HS (2·4+1·2, p=0·016), IC (2·3+0·8, p= 0·001),and NES (2·3+1·2, p=0·025); no statistical differences were detected between MBS and all other types of surgery (p≥ 0·187). The average cost per procedure-related readmission after MBS (£1317+369) was lower compared to HS(£1741+280, p=0·007) but similar to all other types of surgeries (P ≥ 0·055;Fig. 2D).

**Fig. 3:**
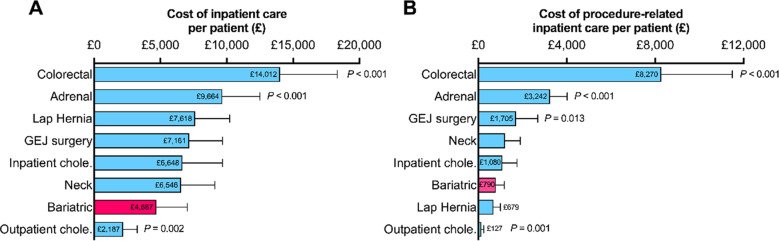
Cost of all (**A**) and procedure-related (**B**) inpatient care per patient over the 5 years post-surgery in each operation type (*n*=100 in each). Bars represent mean ± 95% confidence intervals. Analysed by the Kruskal-Wallis test with Bonferroni-adjusted pairwise comparisons (Mann-Whitney). *P*-values show statistically significant differences between bariatric surgery and comparator surgery

Finally, the average 5-year cost-of healthcare usage per patient for procedure-related readmissions after MBS was £790+337, exceeding only HS and OC and well below the cost for CR (£8,270+ 3218, p<0·001). (Figure 3, Appendix 2).

#### Outpatient Visits

A total of 24,758 outpatient appointments were recorded across all groups. The data received did not allow to precisely differentiate if the visit was procedure related or not, so these episodes were analysed as total visits only. MBS had the second highest overall number of outpatient visits after colorectal surgery (Table 4, Appendix 2). This difference was driven primarily by outpatient visits occurring in the first 2 years postoperatively.

The total cost of outpatient visits after MBS was the highest of all operation types (Table 4, Appendix 2). Conversely, the cost per visit after MBS was lower than for IC and OC, AS, GEJ, HS and NES (*p* < 0·001), and similar to CR (*p* = 1·000). The total cost of outpatient visits per patient was higher for MBS compared to IC and OC (*p* < 0·001), and HS (*p* = 0·002), reflecting the higher number of visits. However, the cost per patient was not significantly different between MBS and the other procedures.

### Five-Year Accident and Emergency Room (A&E) Visits

A total of 2,431 visits to the accident and emergency room were recorded over 5 years post-surgery for all procedures groups. There were no statistically significant differences in emergency room attendance among groups over the 5-year period. However, MBS was associated with lower cost per visit (£100 *±* 6) compared to CR (£139 *±* 6, *p* < 0·001), IC (126 *±* 5, *p* < 0·001), NES (120 *±* 6, *p* < 0·001), and AS (111 *±* 6, *p* = 0·022). The cost per patient was not significantly different between MBS and other procedures (Table 5, Appendix 2).

## Discussion

This study is the first to directly compare perioperative safety, 5-year healthcare use (HU), and costs of MBS with other elective general surgeries. Overall, MBS outcomes were comparable or better. It had lower inpatient and 30-day major complications, fewer 30-day readmissions, and shorter LOS than CR and AS, similar to others.

Over 5 years, MBS showed low all-cause and procedure-related readmission rates. Only 17% of MBS readmissions were procedure-related—significantly less than CR (55%, *p* < 0.001), AS (32%, *p* < 0.001), and GEJ (27%, *p* = 0.003). LOS and hospital costs for readmissions were lower than CR, GEJ, and AS, and similar to AS and cholecystectomy.

A&E visit rates were similar across groups, but per-visit costs were lower after MBS, indicating milder complications. All complication and readmission rates aligned with published benchmarks [7], supporting external validity.

Several studies have compared the costs of surgical vs. non-surgical management of severe obesity. Some reported net savings when using a lifetime time horizon [26], while the majority report similar costs between surgical and non-surgical groups over 4–10 years post-procedure, though results vary across studies [22, 27], with robust evidence of cost-effectiveness for MBS for patients with obesity and/or diabetes [3, 9, 20, 21].

This study is the first to contextualize MBS healthcare use and costs by comparing it to other common, widely accepted surgeries. While these procedures are not alternatives to MBS for patients with obesity or diabetes, they offer a benchmark for evaluating safety and care costs in routine surgical practice.

Previous research has also consistently shown that MBS improves a wide range of obesity-related co-morbidities and decreases mortality [2, 28]. Arguably, the impact of MBS on the health and quality of life of operated patients is at least not inferior to that of the other elective surgeries analysed in this study. Nevertheless, these procedures often receive priority over bariatric surgery in resource allocation, insurance coverage, and public health planning, with their risks widely accepted by patients, providers, and insurers. Yet, MBS —despite targeting a highly prevalent and serious disease—remains underutilized compared to surgeries for conditions of similar or even lower prevalence [7, 10].

Our findings challenge common fears about MBS risks, showing its safety profile is at least comparable to other widely accepted surgical treatments.

This study compared surgical treatments for different diseases and conditions, with inherent differences in baseline disease and demographics between groups. However, intrinsic to the aim of this study is the need to account for various aspects of surgical practice that influence safety and costs, including index disease and baseline characteristics of typical surgical candidates. Thus, in order to reflect real-world practice, we included unselected, consecutive patients who underwent a wide array of standard procedures for each type of surgical treatment, intentionally not matching for baseline characteristics.

Notably, MBS patients had more comorbidities and higher baseline ASA scores, indicating greater postoperative risk than other groups—reasonably excluding bias in favour of MBS.

Several assumptions contribute to misperceptions about MBS risks and costs. People with obesity or diabetes are seen as prone to surgical complications, and, on the other hand, obesity is often viewed as a risk factor—not a life-threatening disease—reducing perceived urgency for surgery. Despite evidence to the contrary, many believe that obesity is entirely reversable through mere lifestyle changes, even when severe [13]. In this context, surgical treatment of obesity, no matter how safe, may be perceived as disproportionately riskier and costlier than diet and exercise. Such perception can mislead decision-making by patients, referring physicians and policymakers.

This study has several limitations. Being limited to hospital data, it does not capture primary care usage after procedures. This may have lead to underestimation of total healthcare costs across specialties and the cost may mostly reflect hospital-based care. Outpatient and A&E visit data lacked sufficient diagnostic detail, preventing distinction between disease- or procedure-related visits and therefore accurate assessment could not be made, possibly a topic for future studies. All bariatric surgeries were performed by one surgeon. FR is an experienced bariatric and metabolic surgeon who has either led or co-led landmark studies in MBS and recently chaired the Lancet commission on defining clinical obesity [29]. Other procedures involved multiple surgeons who have similar clinical seniority and are also leading experts in their respective fields. All surgeries occurred at the same hospital, so results may differ elsewhere; however, comparable post-surgical outcomes suggest this had limited impact. Healthcare use was assessed over 5 years, possibly underestimating longer-term healthcare use due to revisional surgery, but this period is adequate to evaluate safety and complication-related usage. Finally, the period during which the data was gathered may be considered as limitation given the continuous progression of surgical care as well as changing financials in fluctuating economies. However the comparison remains valid since any progress or economic effect will carry over across general surgical specialities.

In conclusion, contrary to widespread perceptions, the results of this study provide evidence that MBS is associated with a relatively low risk of post-operative complications and a 5-year healthcare utilization comparable or better than other elective general surgery procedures. These findings can facilitate evidence-based decision making by patients, referring physicians, and policy makers. 

## Figures and Tables

**Fig. 4:**
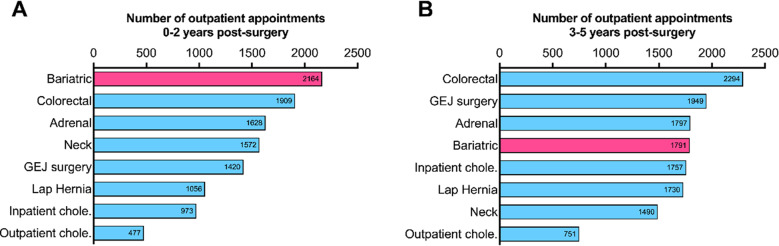
Number of outpatient appointments 0–2 years (**A**) and 3–5 years (**B**) post-surgery in each operation type (*n*=100 in each)

## Data Availability

The data used in this manuscript was provided by NHS digital. As part of the DSA, the original data has been deleted. However, a derived copy of the data containing the data used to generate the results is still available for review. This data is anonymized and derived from the original data in a way that the original data can not be re-engineered or recreated.

## Appendix 2

**Table 3:** Number and cost of 30-day A&E visits

	**Bariatric**	**Adrenal**	**GEJ**	**Colorectal**	**Inpatient Cholecystectomy**	**Lap Hernia**	**Neck **	**Outpatient Cholecystectomy**	
Total number of A&E visits	20	23	13	29	19	10	9	12	
Total cost of A&E visits (£)	1583	2183	1115	3249	2121	983	1018	1452	
Cost per A&E visit (£)	79 ± 17	95 ± 17	86 ± 18	112 ± 14 **	112 ± 18	98 ± 19	113 ± 13	121 ± 21 **	
Data are represented as means ± 95% confidence intervals, or percentages. A&E, accident and emergency. **denotes different from bariatric surgery, *P* < 0.01; *denotes different from bariatric surgery, *P* < 0.05

**Table 4:** Number and cost of outpatient visits (5 years)

	**Bariatric**	**Adrenal**	**GEJ**	**Colorectal**	**Inpatient Cholecystectomy**	**Lap Hernia**	**Neck **	**Outpatient Cholecystectomy**
Total number	3,955	3,425	3,369	4,203	2,730	2,786	3,062	1,228
Total cost (£)	305,971	294,307	273,696	305,029	201,707	216,106	275,759	84,990
Cost per appointment (£)	95 ± 2	109 ± 2 **	108 ± 2 **	98 ± 2	105 ± 3 **	110 ± 3 **	109 ± 2 **	108 ± 4 **
Cost per patient (£)	3,060 ± 605	2,943 ± 599	2,737 ± 599	3,527 ± 602	2,764 ± 630 **	2,641 ± 612 **	3,330 ± 608	1,728 ± 704 **
Data are represented as means ± 95% confidence intervals, or counts. **denotes different from bariatric surgery, *P* < 0.01; *denotes different from bariatric surgery, *P* < 0.05

**Table 5:** Number and cost of A&E visits (5 years)

	**Bariatric**	**Adrenal**	**GEJ**	**Colorectal**	**Inpatient Cholecystectomy**	**Lap Hernia**	**Neck **	**Outpatient Cholecystectomy**	
Total number	296	269	378	373	373	279	248	215	
Total cost (£)	29,525	29,735	37,952	51,982	46,852	29,824	29,740	23,131	
Cost per visit (£)	100 ± 5	111 ± 6 *	100 ± 5	139 ± 5 **	126 ± 5 **	107 ± 6	120 ± 6 **	108 ± 7	
Cost per patient (£)	295 ± 115	297 ± 119	380 ± 114	520 ± 118	469 ± 120	298 ± 119	297 ± 125	231 ± 130	
Data are represented as means ± 95% confidence intervals, or counts. A&E, accident and emergency. **denotes different from bariatric surgery, *P* < 0.01; *denotes different from bariatric surgery, *P* < 0.05									

## References

[1] Colquitt JL, Pickett K, Loveman E, Frampton GK. Surgery for weight loss in adults. Cochrane Database Syst Rev. 2014;2014. 10.1002/14651858.CD003641.pub4. 10.1002/14651858.CD003641.pub4 PMC9028049 25105982

[2] Mingrone G, Panunzi S, De Gaetano A, et al. Metabolic surgery versus conventional medical therapy in patients with type 2 diabetes: 10-year follow-up of an open-label, single-centre, randomised controlled trial. Lancet. 2021;397:293–304. 33485454 10.1016/S0140-6736(20)32649-0

[3] Schauer PR, Bhatt DL, Kirwan JP, et al. Bariatric surgery versus intensive medical therapy for diabetes — 5-Year outcomes. N Engl J Med. 2017;376:641–51. 28199805 10.1056/NEJMoa1600869 PMC5451258

[4] Sandblom G, Videhult P, Crona Guterstam Y, Svenner A, Sadr-Azodi O. Mortality after a cholecystectomy: a population‐based study. HPB. 2015;17:239–43. 25363135 10.1111/hpb.12356 PMC4333785

[5] Robertson AGN, Wiggins T, Robertson FP, et al. Perioperative mortality in bariatric surgery: meta-analysis. Br J Surg. 2021;108:892–7. 34297806 10.1093/bjs/znab245

[6] Perioperative safety in the longitudinal assessment of bariatric surgery. N Engl J Med. 2009;361:445–54. 19641201 10.1056/NEJMoa0901836 PMC2854565

[7] Kazaure HS, Roman SA, Sosa JA. Association of postdischarge complications with reoperation and mortality in general surgery. Arch Surg. 2012;147:1000. 23165614 10.1001/2013.jamasurg.114

[8] Padwal R, Klarenbach S, Wiebe N, et al. Bariatric surgery: a systematic review of the clinical and economic evidence. J Gen Intern Med. 2011;26:1183–94. 21538168 10.1007/s11606-011-1721-x PMC3181300

[9] Hoerger TJ, Zhang P, Segel JE, Kahn HS, Barker LE, Couper S. Cost-effectiveness of bariatric surgery for severely obese adults with diabetes. Diabetes Care. 2010;33:1933–9. 20805271 10.2337/dc10-0554 PMC2928336

[10] Borisenko O, Colpan Z, Dillemans B, Funch-Jensen P, Hedenbro J, Ahmed AR. Clinical indications, utilization, and funding of bariatric surgery in Europe. Obes Surg. 2015;25:1408–16. 25528567 10.1007/s11695-014-1537-y PMC4498278

[11] Dixon JB. Regional differences in the coverage and uptake of bariatric–metabolic surgery: A focus on type 2 diabetes. Surg Obes Relat Dis. 2016;12:1171–7. 26948939 10.1016/j.soard.2015.11.027

[12] Rubino F, Puhl RM, Cummings DE, et al. Joint international consensus statement for ending stigma of obesity. Nat Med. 2020;26:485–97. 32127716 10.1038/s41591-020-0803-x PMC7154011

[13] O’Keeffe M, Flint SW, Watts K, Rubino F. Knowledge gaps and weight stigma shape attitudes toward obesity. Lancet Diabetes Endocrinol. 2020;8:363–5. 32142624 10.1016/S2213-8587(20)30073-5

[14] Goodchild S. Weight-loss surgery can ‘ruin patients’ quality of life’, warns leading doctor. 06/07/2015. 2015; published online July 6. https://www.independent.co.uk/life-style/health-and-families/health-news/weightloss-surgery-can-ruin-patients-quality-of-life-warns-leading-doctor-10367509.html Accessed 8 May 2023.

[15] Naish J. Are surgeons hiding the deadly risks of obesity surgery? 10/06/2014. 2014; published online June 10. https://www.dailymail.co.uk/health/article-2653312/Are-surgeons-hiding-deadly-risks-obesity-surgery.html Accessed 8 May 2023.

[16] Stolberg CR, Hepp N, Juhl AJA, Juhl BCD. Primary care physician decision making regarding referral for bariatric surgery: a national survey. Surg Obes Relat Dis. 2017;13:807–13. 28336199 10.1016/j.soard.2017.02.002

[17] Sarwer DB, Ritter S, Wadden TA, Spitzer JC, Vetter ML, Moore RH. Physicians’ attitudes about referring their type 2 diabetes patients for bariatric surgery. Surg Obes Relat Dis. 2012;8:381–6. 22386926 10.1016/j.soard.2011.12.013 PMC3865449

[18] Tork S, Meister KM, Uebele AL, et al. Factors influencing primary care physicians’ referral for bariatric surgery. JSLS. 2015;19:e201500046. 10.4293/JSLS.2015.00046 PMC4539491 26390524

[19] Sarwer DB, Ritter S, Wadden TA, Spitzer JC, Vetter ML, Moore RH. Attitudes about the safety and efficacy of bariatric surgery among patients with type 2 diabetes and a body mass index of 30–40 kg/m2. Surg Obes Relat Dis. 2013;9:630–5. 23260805 10.1016/j.soard.2012.10.007 PMC3866014

[20] Gulliford MC, Charlton J, Prevost T, et al. Costs and outcomes of increasing access to bariatric surgery: cohort study and cost-effectiveness analysis using electronic health records. Value Health. 2017;20:85–92. 28212974 10.1016/j.jval.2016.08.734 PMC5338873

[21] Boyers D, Retat L, Jacobsen E, et al. Cost-effectiveness of bariatric surgery and non-surgical weight management programmes for adults with severe obesity: a decision analysis model. Int J Obes. 2021;45:2179–90. 10.1038/s41366-021-00849-8 PMC8455321 34088970

[22] Neovius M, Narbro K, Keating C, et al. Health care use during 20 years following bariatric surgery. JAMA. 2012;308:1132. 22990272 10.1001/2012.jama.11792

[23] Gasoyan H, Tajeu G, Halpern MT, Sarwer DB. Reasons for underutilization of bariatric surgery: The role of insurance benefit design. Surg Obes Relat Dis. 2019;15:146–51. 30425002 10.1016/j.soard.2018.10.005 PMC6441615

[24] Chhabra KR, Fan Z, Chao GF, Dimick JB, Telem DA. The role of commercial health insurance characteristics in bariatric surgery utilization. Ann Surg. 2021;273:1150–6. 31714318 10.1097/SLA.0000000000003569

[25] Dindo D, Demartines N, Clavien P-A. Classification of surgical complications: a new proposal with evaluation in a cohort of 6336 patients and results of a survey. Ann Surg. 2004;240:205–13. 15273542 10.1097/01.sla.0000133083.54934.ae PMC1360123

[26] Borisenko O, Adam D, Funch-Jensen P, et al. Bariatric surgery can lead to net cost savings to health care systems: results from a comprehensive european decision analytic model. Obes Surg. 2015;25:1559–68. 25639648 10.1007/s11695-014-1567-5 PMC4522026

[27] Smith VA, Arterburn DE, Berkowitz TSZ, et al. Association between bariatric surgery and long-term health care expenditures among veterans with severe obesity. JAMA Surg. 2019;154:e193732. 31664427 10.1001/jamasurg.2019.3732 PMC6822094

[28] Albaugh VL, Kindel TL, Nissen SE, Aminian A. Cardiovascular risk reduction following metabolic and bariatric surgery. Surg Clin North Am. 2021;101:269–94. 33743969 10.1016/j.suc.2020.12.012

[29] Rubino F, Cummings DE, Eckel RH, et al. Definition and diagnostic criteria of clinical obesity. Lancet Diabetes Endocrinol. 2025;13:221–62. 39824205 10.1016/S2213-8587(24)00316-4 PMC11870235

