# Correction: Post-Operative Safety Outcomes and 5-Year Healthcare Utilisation After Bariatric Surgery vs. Other Elective Types of Surgical Treatments – A Retrospective Observational Cohort Study

**DOI:** 10.1007/s11695-026-08678-2

**Published:** 2026-04-18

**Authors:** Ghassan Chamseddine, Robert David McIntyre, Spyros Panagiotopoulos, A. Caroline Rudisill, James Casella, Lidia Castagneto-Gissey, Avril Chang, Gabriele Galata, Amyn Haji, Asif Haq, Laurent Genser, Geltrude Mingrone, Ameet Patel, Angelo Salerno, Daniele Tassinari, Francesco Rubino

**Affiliations:** 1 Department Bariatric/Metabolic & Minimally Invasive Surgery, King’s College Hospital NHS Foundation Trust, London, UK; 2 School of Sport, Exercise & Applied Science, St Mary’s University Twickenham London, London, UK; 3 Department of Health Promotion Education & Behavior, Arnold School of Public Health, University of South Carolina, Columbia, USA; 4 Department of General Surgery, Ospedale dei Castelli, Rome, Italy; 5 Department of Surgical Sciences, Sapienza University of Rome, Rome, Italy; 6 Department of Endocrine & General Surgery, King’s College Hospital NHS Foundation Trust, London, UK; 7 Department of Colorectal Surgery, King’s College Hospital NHS Foundation Trust, London, UK; 8 Hepato-Biliary & Pancreatic Surgery, Pitié-Salpêtrière University Hospital, Sorbonne University Assistance Publique-Hôpitaux de Paris, Geltrude Mingrone, Paris, France; 9 Catholic University of the Sacred Heart, Milan, Italy; 10 School of Cardiovascular & Metabolic Medicine & Sciences, King’s College London, London, UK; 11 IRCCS Ospedale Galeazzi Sant’Ambrogio, Milan, Italy

**Correction: Obesity Surgery**

**https://doi.org/10.1007/s11695-026-08584-7**

The original article has been updated to add a missing funding statement.

